# Staff Perspectives of Mass Casualty Incident Preparedness

**DOI:** 10.7759/cureus.15858

**Published:** 2021-06-23

**Authors:** Mary E Moran, Jacob R Zimmerman, Amelia D Chapman, Derek A Ballas, Nathan Blecker, Richard L George

**Affiliations:** 1 Research, Sponsored Programs, and Innovation, Summa Health, Akron, USA; 2 Surgery, Division of Trauma, Summa Health, Akron, USA; 3 Medicine, College of Health Sciences, University of Kentucky, Lexington, USA; 4 Medicine, Ohio University Heritage College of Osteopathic Medicine, Warrensville Heights, USA; 5 Medicine, Summa Akron City Hospital, Summa Health System, Akron, USA; 6 Obstetrics and Gynecology, Summa Health, Akron, USA; 7 Surgery, Northeast Ohio Medical University, Rootstown, USA

**Keywords:** quality improvement projects, qualitative, mass casualty incident, emergency preparedness, emergency response plan, in situ simulation

## Abstract

Introduction

Mass casualty incidents (MCI) are low-frequency, high-risk events that disrupt the day-to-day operations of medical centers. Day-to-day protocols are insufficient for effectively managing MCI events, creating a need to develop and test MCI-specific protocols. The aim of this project was to utilize interviews to gain insight into staff experience and perspective on MCIs and their institution’s response plans.

Methods

Staff members who participated in an MCI drill were asked semi-structured interview questions regarding their perspectives on their current priorities, the information needed to perform their role, and their greatest concerns about an MCI. This quality improvement (QI) project utilized a qualitative methodology to thematically organize the results of the staff responses.

Results

A total of 64 staff members with various levels of patient care experience were interviewed to reach thematic saturation. The use of staff interviews helped to identify the four primary themes that emerged, which were: 1) process, 2) supplies and resources, 3) communication, and 4) roles. Furthermore, each theme also included a number of subthemes.

Conclusions

This project demonstrated the importance of staff experiences related to MCI simulation training and preparedness, which may be useful for future training and emergency response planning. Additionally, the results may be helpful for other institutions when building a robust MCI simulation training program or designing an emergency response plan.

## Introduction

According to the World Health Organization, the definition of a mass casualty incident (MCI) is “an event which generates more patients at one time than locally available resources can manage using routine procedures” [1]. The number of disasters affecting communities, which includes natural disasters and mass shootings, has increased over time [2-4]. The Federal Emergency Management Agency (FEMA) reported a total of 852 natural disasters from 2015 to 2020, which average to 142 per year [4]. There was a 205% increase in natural disasters from 2019 to 2020, with 101 and 308 disasters being reported, respectively [4]. Incident rates of mass shooting events have increased over the last 20 years as well, with only one being reported in the year 2000 and 10 in 2019 [3]. In addition, the number of victims of MCIs has increased [2-3]. Evidence of substantial increases in MCIs and the number of injured persons has raised concerns regarding the importance of the issue and leads to concerns of a public health crisis; therefore, demonstrating the need for health care systems to increase preparedness.

Public health professionals have identified MCIs from gun violence as a public health concern [5]. This may have contributed to the concerns for health care systems to increase preparedness. Professional organizations, including the American College of Surgeons (ACS), have come forward to declare an obligation of healthcare leaders and providers to take an active role in the interdisciplinary preparation of MCIs [6-7]. Because hospitals and their staff will be responsible for treating and managing injured victims of MCIs, these institutions have an imperative role within MCI preparedness [8-11], which could result in subsequent lives saved [12].

Due to the unpredictable nature of MCIs, day-to-day protocols are insufficient to effectively manage the evolution of an MCI, specifically, the inability to manage severe resource constraints [8,11-13]. Normal hospital procedures do not prepare staff for patient surges that may exceed the available resources [8,11-13]. Routine Emergency Department (ED) triage is insufficient during an MCI, as it requires significant time and would delay throughput [8-11]. Furthermore, surgical services such as the Operating Room (OR) and Post-Anesthesia Care Unit (PACU) may need to make modifications to their day-to-day protocols. The workflow may need to be adapted, such as canceling elective cases, performing “damage control” operations, or turning the PACU into a holding area [8,11,14]. Hospital-wide, other strategies include expediting discharges [8,11], changing physical space to increase inpatient capacity [11,14], and using outpatient areas and staff to accommodate MCI victim influx [14]. To develop an effective emergency response plan, hospitals should focus on creating flexible protocol standards separate from day-to-day operations to account for an array of incidents and injuries [8,11-13,15].

Role of simulation

Simulation training drills should be included when creating a response plan for MCIs, as this training not only provides an opportunity for multidisciplinary feedback but allows for staff members to test the emergency response plan [13,16-27]. There are different types of simulation, which include skill development in a controlled environment or practice in the clinical space, called *in situ* simulations.

Simulation has been found to be an effective training model to increase MCI preparedness [13,16-29]. Simulation training can impact MCI preparedness by improving clinical decision making, [24] competency [28], protocols [29], and preparation and confidence [27]. Furthermore, *in situ* simulation can specifically impact MCI-related perceptions of preparedness [13,21], ED triage accuracy [17-18,22], reduced triage time [18], and accuracy regarding treatment decisions [19]. While testing an emergency response plan *in situ* sounds challenging, it has demonstrated benefits in knowledge retention compared to didactic learning models [26]. Overall, *in situ* MCI simulations provide a realistic experience for healthcare providers to practice critical skills required during an MCI, as well as provide feedback on how to improve current emergency response protocols.

The purpose of this project was to better understand staff perspectives of MCI preparedness after participating in MCI simulation drills at a tertiary referral center and Level I trauma center. It was submitted to the institutional review board and qualified for exemption status as a quality improvement (QI) project.

## Materials and methods

Study participants and design

This study took place from February 2019 to June 2019 at an ACS-verified Level I Trauma Center in the Midwestern United States that treats approximately 2,000 trauma admissions and 85,000 ED visits annually. The participants in this study were staff members of various levels (i.e., medical students, residents, attending physicians, ED registered nurses, advance practice providers, etc.) and departments, such as trauma, ED, intensive care unit, OR, PACU, protective services, ancillary services (i.e., respiratory therapy, occupational therapy, physical therapy), and environmental services. These participants were selected using convenience sampling and were asked to participate in the semi-structured interviews after completing the *in situ *MCI simulation drill. This study was reviewed by the institutional review board administrator and was determined that the study met the criteria for a quality improvement project, did not require the full institutional review board review, and was approved as a quality improvement project.

The standardized *in situ *MCI drills were developed using the same stem, the number of patients (eight to 10), and unannounced patient presentation to test the emergency response plan for multiple gunshot wound victims. Each drill was approximately 30 minutes in duration and consisted of two components; a 20-minute *in situ *MCI simulation followed by a 10-minute debrief. The simulation drills were limited to a specific time and department to enable staff participation in a drill. The focus was on individual clinical areas depending on the scope of the drill, including ED, OR, and PACU. Limited resources were required due to the small number of simulated patients and the brevity of the drill; furthermore, the design of the *in situ *simulation assisted in the application of the emergency response protocols within the clinical space while ensuring a safe clinical environment. Participants were expected to respond to the *in situ *MCI simulation as they would respond to a true mass casualty event. Staff members from the MCI drill design team followed participants to observe and evaluate the process flow of the emergency response plan. The MCI drill design team included members from the following departments: Emergency Preparedness, Surgery, Emergency Medicine, Nursing, Registration, Materials Management, Medical Education (Simulation), and Research.

After a participant was finished with their task in the *in situ* MCI simulation and transitioned the “victim” to a new care provider or new area, the drill team observer asked semi-structured interview questions, which were audio-recorded. The semi-structured interview was developed by content experts and approved by the multidisciplinary MCI drill design team, and included questions about the participant’s day-to-day clinical duty, role changes during an MCI, and questions related to the *in situ *MCI simulation drill. Overall, questions sought to answer what the current priorities are for each participant, what information the participant needed to do their job and how they would obtain it, steps followed to provide clinical care, how participants interact with leadership, and what their greatest concern is about an MCI.

Data analysis and trustworthiness strategy

This QI project utilized staff perspectives via semi-structured interviews and qualitative methodology to thematically organize the results of the staff responses. One of the non-clinical co-investigators transcribed the interviews verbatim and removed names to preserve participant anonymity. Participants were not identified by their names during the semi-structured interviews, which reduced the risk of identification, and roles were general enough to enable anonymity.

The lead investigator and one co-investigator reviewed the transcriptions and used qualitative research software NVivo Plus version 12 (QSR International, Melbourne, Australia) to organize and track interview transcripts and thematically code the data. As there is a dearth of literature regarding staff perspectives on *in situ* MCI simulation drills and emergency preparedness, the QI project sought to explore the wider themes that staff reported. Codes were assigned to staff responses through iterative discussion and refinement, which eventually merged into categories. Categories were then merged into themes and subthemes that captured the essence of the experience described by the participants. In total, 64 post-MCI simulation staff interview transcripts were analyzed for quantitative study when thematic saturation was reached.

Triangulation was used as a way to reduce the risk of bias as a data trustworthiness strategy. First, the two researchers read each of the transcribed interviews independently. Second, the researchers sat together to code the transcripts and had discussions regarding the codes before reaching a complete agreement on the appropriate codes. Third, the researchers began grouping the codes to create categories, subthemes, and themes.

## Results

**Figure 1:**
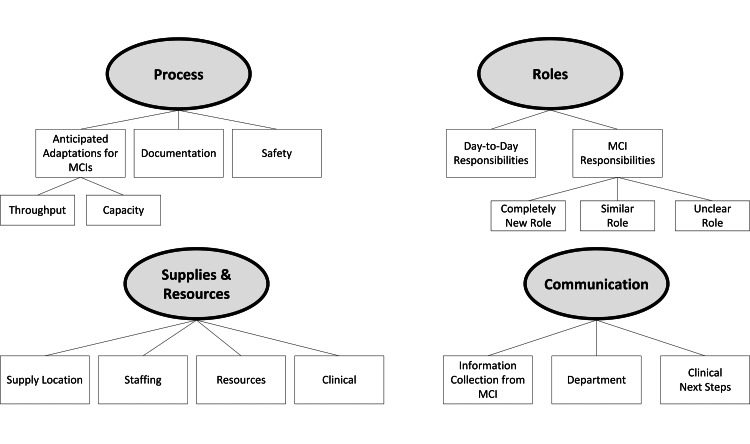
Concept Map of Staff Interviews Note. MCI = mass casualty incidents.

The themes that emerged from qualitative analysis are displayed in Figure 1. The four primary themes included 1) process, 2) supplies and resources, 3) communication, and 4) roles. Furthermore, there were a number of specified subthemes that prevailed during analysis (see Table 1). These four common themes and their additional subthemes are explored below.

**Table 1:** Data Analysis and Categorization Note. ER = emergency room, MCI = mass casualty incident.

Theme	Subtheme (if applicable)	Data
	Anticipated Adaptations for MCIs - Throughput	How many would come in, we mainly have our plan set up […], but there is only 25 to 30 beds there.
Process	Anticipated Adaptations for MCIs - Capacity	Typically we are pretty full up here, our capacity is around 90 percent […] my main concern is not having beds.
	Safety	Physically losing patients in a space, overcrowding the ER
	Documentation	Documentation would come after patient care, we [would] have paper recording.
	Supply Location	we have bandage carts that we call for from distribution
Supplies &	Staffing	We can pull from same day surgery and […] contact the nursing supervisor to bring available staff to care for patients if we got a large volume.
Resources	Resources	I have the policy [emergency response plan] and I can also refer to the charge nurse or supervisors.
	Clinical	If they were all more critical I would worry more about respiratory supplies, ventilators.
	Information Collection from MCI	We would need to know what the MCI was, if the hospital is locked down, if we need to be safe in the hospital and on our unit.
Communication	Department	Distribution is supposed to bring up a cart of additional supplies for an MCI.
	Clinical Next Steps	Patients they bring up to the floor, we have to assess quickly, determine vital signs, hook them up to telemetry […] and then try to find rooms for them as quickly as we can.
	MCI Responsibilities –Similar Role	unless there’s an original charge nurse that [assigns you to] patients coming in, the rest of us will take care of the existing patients.
Roles	MCI Responsibilities – Completely New Role	We kind of run as ancillary staff [during an MCI], and we report to our supervisors, in this incident we were paged down to the ER if we were not doing direct patient care, and we were given a job role.
	MCI Responsibilities – Unclear Role	I do not think everybody’s got their [MCI] roles yet, and it may be making them a little more confused.
	Day-to-Day Responsibilities	Staff roles were identified

MCIs require changes to processes and there were three subthemes that emerged within this theme: 1) anticipated adaptations for MCIs, 2) safety, and 3) documentation. Within the subtheme of anticipated adaptations for MCIs, interviewed staff expressed the need for adaptations to deal with challenges, like potential clinical supply shortages and the anticipation of large patient surges by stating, *“I would start ripping off bed sheets and tie them around people’s legs.”* It is clear that staff members began to prepare for the changes that may ensue due to an MCI. Additionally, there were two categories that emerged from the anticipated adaptations for MCIs, throughput, and capacity. Staff consciously recognized the need for a shift in the process when an MCI is called, specifically related to throughput and capacity limitations, *“How many would come in [. . ]. there is only 25 to 30 beds there [. . .] plus there are patients already here who are already in place, so I know they call mass casualty, and people come down and take patients to other floors, but that is a whole other dance.”* Limited capacity also creates triage safety concerns, which was another subtheme from the analysis.

A general concern for the safety of staff and patients was voiced: *“All the patients [should] be safely accounted for and given that it is a natural disaster and not a man-made, safety of incoming patients and staff is priority.” *Accounting for patients during patient triage was discussed, as there may be a risk of a perpetrator presenting to the hospital to conflict more injury or as a patient, which can put all staff and patients in danger. Triage location was included within safety, as knowing the location of patient cohorts with higher acuity injuries allows for more attentive care by continuously monitor and re-assessing patient triage cohorts.

The final subtheme that emerged within the process was documentation. Staff discussed that methods of documentation could vary given differing levels of urgency and access to documentation technology or the use of paper and writing utensils. *“Documentation would come after patient care, we have paper recording and we have nursing personnel who are assigned to document as needed, but all orders would be verbal.” *When an MCI occurs, the process must be manipulated in accordance with the development of the incident, urgency, and safety. While this documentation is one aspect of an MCI that requires adjustment, other areas may also experience changes to the process or availability.

Supplies and resources

As an MCI does not fit into day-to-day protocols, staff expressed concerns about the limitations of supplies and resources. The four subthemes that emerged within this theme of supplies and resources were: 1) supply location, 2) staffing, 3) resources, and 4) clinical. Supply location included MCI-specific materials, some of which were kept in a separate location from the day-to-day materials. One example was the trauma cart, which contained vests to identify key leaders within the ED and an abundance of trauma supplies. Staff expressed the following concerns regarding supply location: *“So we have bandage carts that we call for from distribution [an off-site location], we can call them and they will bring up extra supplies, we have extra beds down in the bays, we have 20 of the folding PVC beds that we are able to use, most of it comes up on the distribution carts.”* Those interviewed expressed concern for not only shortage of supplies, but also a shortage of staff when asked about MCI preparedness. When discussing staffing concerns, a participant noted, *“We can pull from same-day surgery and send out the alert to get additional staff here, we can also contact the nursing supervisor to bring available staff to care for patients if we got a large volume.” *This indicated the staff member’s knowledge of the emergency response plan and the departmental needs.

Ease of access to resources and reference materials was cited as a priority for staff members to help clarify role responsibilities and protocols during an MCI. One participant stated:* “I have the policy that I can look up on [health care organization’s staff website] for the disaster code yellow policy and I can also refer to the charge nurse or supervisors.” *Because MCIs are so varied and unpredictable, staff are able to access information when confusion or doubt arises. Additional areas of concern for staff were the internal transportation measures during an MCI, such as wheelchairs for the walking wounded, and a potentially limited supply of linens depending on the size of an MCI. The clinical subtheme referred to trauma-specific supplies necessary for patient care during an MCI, which included respiratory care, blood, chest tubes, wound care, and vital sign measurements.

Communication

The next theme was communication, which included three subthemes: 1) information collection from the MCI, 2) department, and 3) clinical next steps. One staff shared,* “We would need to know what the mass casualty incident was, if the hospital is locked down, if we need to be safe in the hospital and on our unit.” *This response demonstrated the knowledge that depending on the MCI scenario, the patient injuries and needs may be different; therefore, the emergency response plan needs to allow for flexibility. Within the clinical next steps subtheme, nursing staff from one of the inpatient nursing units shared some of their expectations, *“Patients they bring up to the floor, we have to assess quickly, determine vital signs, hook them up to telemetry monitors [. . .] and then try to find rooms for them as quickly as we can.” *This inpatient nurse was prepared to change the day-to-day process for receiving patients on the unit and was already planning for challenges associated with limited patient rooms, which would typically be exacerbated during an MCI event. Communication at the department level was important due to the method of contacting specific departments.

There were three specific departments that were discussed during the semi-structured interviews. One staff member specifically stated their first step would be to contact the blood bank. The Hospital Incident Command System (HICS) was referenced by one staff, *“I am going through the ED commander who is going through the command center to get me what I need,”* indicating the staff’s knowledge that additional resources may be available if the MCI was large enough and there was a HICS initiated. Another department that was specifically mentioned was Materials Management where one staff shared, *“Distribution [Materials Management] is supposed to bring up a cart of additional supplies for a mass casualty, we could also contact the nursing supervisor and distribution.”* Based on the information collected, staff members were knowledgeable on the adapted processes outlined in the emergency response plan regarding communicating their needs during an MCI.

Roles

Many staff shared the importance of their roles; a theme which was further broken down into two subthemes, day-to-day responsibilities, and MCI responsibilities. A number of staff members felt that their role would not change if an MCI were to occur. Interestingly, of staff who commented on the anticipated changes of their roles during an MCI, three categories of MCI roles emerged: 1) similar role, 2) completely new role, and 3) unclear role. For some staff members, their day-to-day responsibilities carry more urgency than usual in an MCI, and thus they perform a role similar to one they perform regularly should an MCI arise. Nursing assignments may remain the same, as in continuing to treat pre-existing patients, while others may be reassigned to care for the presenting patients: *“Unless there’s an original charge nurse that [assigns staff to] take care of the patients coming in, the rest of us will take care of the patients already here.”*

Others may take on an entirely new role as compared to their day-to-day responsibilities to meet the immediate demands and reduce strain on the institution’s response to the MCI. Available physical therapy staff can be reorganized as patient transporters, as described in one of the staff interviews:* “We [physical therapists] kind of run as ancillary staff, and we report to our supervisors, in this incident we were paged down to the ER if we were not doing direct patient care, and we were given a job role.”* As an MCI can have unclear details and developments, roles at a facility for certain staff may be unclear, especially when staff members are caught off-guard. One staff member shared examples of shift change, those who have not received role assignments for the day or are unprepared to activate and execute their MCI role. The confusion regarding staff roles may be dependent upon the unpredictable nature of MCIs, which is one of the major challenges with MCI preparedness. This challenge extends beyond the staff roles and into other areas.

## Discussion

The purpose of this project was to better understand staff perspectives of MCI preparedness after participating in MCI simulation drills at a tertiary referral center and Level I trauma center. Existing literature is either anecdotal [7-12,14-15] or uses quantitative methods [13,19-20,22,24,26-29] to demonstrate the impact of simulation on emergency preparedness. Findings from this study help elaborate on the staff perspectives, adding to the body of research, and may impact the planning and implementation of emergency preparedness training at other health care institutions. While previous literature clearly supports the need to change day-to-day responsibilities, protocols, and have awareness of resource scarcity [8,11-13,15] the semi-structured interviews provided an opportunity for staff perspectives regarding MCI preparedness and simulation training to be explored. The four themes: 1) process, 2) supplies and resources, 3) communication, and 4) roles are discussed in relation to the existing literature below.

One noteworthy finding was in relation to the roles and the change from day-to-day responsibilities for staff. While the concept of changes in responsibility has been published [8-9,14-15], most are not reporting staff perspectives. There are limited qualitative studies available that explore the perspectives of staff; two such studies exist with the focus on student experiences related to MCI simulations as a training model [23,30]. Whereas this study explored the knowledge of staff perspectives related to the emergency response plan. Additionally, the use of ancillary staff to completely stop their day-to-day responsibilities in order to fulfill MCI-specific responsibilities has not been widely acknowledged in other studies. This result demonstrates the importance of *in situ* MCI simulation in order to identify novel and unexpected utilization of staff to fulfill MCI responsibilities. The emergency response plan would not likely account for the reallocation of physical therapists without testing the response plan using simulation. Responsibilities are not the only changes that occur during an MCI. Resources, such as the number of staff available, can impact the successful implementation of an emergency response plan.

Resource scarcity has been discussed at length within MCI literature is integrated into emergency response plans, including general supplies [8,14], hospital space [8,10-11,15], surgical services space, and time [8,10], and staff [14,27,29]. Similarly, within this study staff demonstrated their extensive awareness of resource scarcity by sharing their knowledge regarding the location of the emergency response plan and the high likelihood that their resources could quickly deplete. Additionally, the staff knew the internal contact to receive auxiliary supplies from an off-site location. The ability of staff to quickly recall this information indicates the level of saturation the MCI training and simulation drills have had on their memory. While there is no need to utilize the emergency response plan or know whom to contact regarding off-site supplies on a daily basis, the staff indicated the additional resources with ease.

Communication is an emphasis of any health care system and MCIs exacerbate the importance of effective communication. Previous literature highlights the different levels of communication including, staff-level regarding patient care [18,23], leadership level [14], and the larger system or infrastructure [7-8]. Staff from this study shared the various aspects of communication, but collecting information from the MCI was remarkable and was not discussed in previous studies. Receiving information from the scene of the MCI would better prepare the hospital for the types of injuries that may present. However, the staff highlighted the importance of knowing whether the hospital or nursing unit needs to be “locked down” due to the potential threat of additional violence or hazardous material contamination.

The processes that were required to change and effectively manage an MCI seem to be innumerable. The scenes depicted of MCI events across the country display chaos and confusion. It was clear that staff felt the pressure and anticipation of exorbitant patient surges that would make the typical documentation process impossible. Information regarding the significant impact of surges has been reported related to triage [7-8], surgical services [8,14], and general information on surge capacity [10,15]. Interestingly, this literature did not discuss the impact of patient surges on documentation. Within this study, staff shared their expectation of taking notes on paper and after the patient surge subsided, document in the electronic medical record.

Some of the staff shared their experiences with barriers when transitioning from day-to-day protocols into MCI protocols. The first theme highlighted the staff’s extensive knowledge and previous training required to locate and obtain MCI supplies. As noted by participants, bandage carts critical for an MCI are stored in off-site locations, making them more difficult to obtain. Staff must know where supplies are stored as well as how to efficiently and effectively communicate the situation to another department to deliver those necessary supplies. These components require MCI-specific training and staff education before an MCI occurs. Unprepared staff can lose valuable time locating this information and waiting for necessary supplies to arrive during critical windows of patient care, leading to poorer outcomes. The first theme also highlighted the importance of knowing which departments are available as extra primary and ancillary aides. Proper training for all employees of MCI protocol and equipment storage may aid staff to better prepare for an MCI.

The importance of process and communication were emphasized due to their potential impact on maximizing care delivery during critical patient care windows. Through training and simulation practice, participants could better anticipate what changes were expected of them during an MCI, which increased the preparedness of participants and increased process efficiency. This impact was notable during capacity and throughput changes, which would allow for the treatment of larger volumes of patients if a patient surge were to occur. Communication with critical departments such as the blood bank to get essential supplies, the hospital incident command system to coordinate responses, and the distribution to get supplies were major events that could greatly impact MCI outcomes if not performed correctly.

Limitations of the study

Limitations of this QI project include those inherent to qualitative methodologies and some that were specific to this project. A general limitation includes the inability to generalize qualitative results to other institutions due to the small sample size. While the results cannot be generalized it does not mean that other institutions cannot find value in the findings, which may help to guide emergency preparedness or *in situ* MCI simulation development. The need to expand communication, day-to-day responsibilities, processes, and resources in order to effectively and efficiently respond to an MCI is not unique to our institution. There were two risks of bias associated with the results of this study. One, staff experience, including potential certifications related to emergency preparedness, may have impacted the results of the staff responses and was unaccounted for during the analysis of the study. Two, there were only two investigators to code and analyze the data, when three would have been preferred in order to have a majority consensus.

## Conclusions

This QI project shared the results of staff perceptions regarding emergency preparedness and *in situ* simulation to enhance preparedness at a Level I Trauma Center in the Midwestern United States using a qualitative analysis. Staff self-reported perceptions on training and *in situ* MCI simulation drills assisted with identifying areas of consideration for MCI preparedness including the location of MCI supplies and resources, understanding MCI-specific roles and processes, and the importance of communication. This project can be used as a model for developing and troubleshooting MCI and emergency management protocols in tertiary care hospitals. The staff perceptions gathered via interviews are aspects that must be considered and accounted for when creating a more robust emergency response plan. Future work is to ensure that staff perspectives are integrated into new staff onboarding as well as continuous emergency preparedness training.
